# Protecting the Ketone: Preparation of Ethyl Acetoacetate Ethylene Ketal and Its Relevance to Medicinal Chemistry

**DOI:** 10.7759/cureus.90222

**Published:** 2025-08-16

**Authors:** Amy Avakian

**Affiliations:** 1 Diagnostic Radiology, Southern Hills Hospital, Las Vegas, USA; 2 Diagnostic Radiology, Ross University School of Medicine, Florida, USA

**Keywords:** 4-diphenyl-3-buten-2-one, biochemistry, carbonyl, chemistry, ethyl, general pharmacology, ketone, medicinal chemistry, molecular pharmacology

## Abstract

This experiment demonstrates the protection of the ketone carbonyl group in ethyl acetoacetate by converting it into an ethylene ketal using ethylene glycol and p-toluenesulfonic acid as a catalyst. The reaction was conducted under reflux in a Dean-Stark apparatus to drive the equilibrium toward ketal formation by removing water. Subsequent purification and nuclear magnetic resonance (NMR) analysis confirmed 92.05% product purity and a yield of 53.1%. This process supports fundamental strategies in medicinal chemistry where functional group protection is critical for the synthesis of complex drug molecules, including steroids and antibiotics.

## Introduction

The multistep synthesis of 4,4-diphenyl-3-buten-2-one, a type of conjugated enone and a synthetic chalcone derivative, illustrates a classic example of functional group manipulation in organic chemistry. A key challenge in such syntheses is chemoselectivity, the ability to modify one functional group in the presence of others. Protecting groups are temporary modifications used to shield reactive functional groups from undesired interactions during multistep transformations. Effective protecting groups must be introduced and removed under mild conditions and must not interfere with other synthetic steps [1].

In this experiment, the ketone group in ethyl acetoacetate is protected through the formation of an ethylene ketal. This step is critical because ketones are more reactive electrophiles than esters and would otherwise be the preferred site of nucleophilic attack during subsequent reactions, such as Grignard additions. By masking the ketone, the ester moiety remains accessible for selective transformation. This strategic use of protecting groups is essential not only in academic synthesis but also in pharmaceutical and biomedical chemistry.

Carbonyl protection plays a vital role in drug development, particularly in the synthesis of corticosteroids, macrolide antibiotics, and certain chemotherapeutics. These multistep syntheses often require careful sequencing of reactions, where selective protection allows for controlled molecular construction. The techniques demonstrated here, including equilibrium control via Dean-Stark apparatus and nuclear magnetic resonance (NMR)-based purity analysis, reflect real-world procedures used in medicinal chemistry and process-scale drug manufacturing [2,3].

## Technical report

Period 1: Protection of the carbonyl group

The initial step in the synthesis involved the formation of the ethylene ketal of ethyl acetoacetate, a commonly employed protecting group in organic synthesis. This reaction was carried out by combining the ketoester with ethylene glycol in the presence of p-toluenesulfonic acid (TsOH), which served as an acid catalyst. Due to its higher electrophilicity, the ketone carbonyl of ethyl acetoacetate preferentially reacted with ethylene glycol over the ester carbonyl, resulting in ketal formation.

The reaction proceeded under equilibrium conditions where water is a byproduct, potentially limiting product formation. To drive the equilibrium toward the desired ketal, it was necessary to continuously remove water from the system. This was achieved using a Dean-Stark apparatus, which facilitates azeotropic distillation. When the mixture was heated, the vapor formed a condensate containing both water and toluene. Upon condensation, the two liquids separated within the trap, with water accumulating at the bottom due to its higher density. Once the liquid level reached the side arm of the trap, the toluene-rich upper layer recycled back into the reaction flask, effectively removing water and shifting the equilibrium toward product formation [4].

Period 2: Isolation of ethyl acetoacetate ethylene ketal

Following the completion of the reflux and water removal, the next step was to isolate the ketal by eliminating residual toluene via simple distillation. The crude reaction mixture was transferred to a three-necked round-bottom flask fitted for distillation. The apparatus joints were properly greased to ensure a secure seal, and boiling chips were added to promote smooth boiling. The system was gently heated with a heating block set to 40°C, and distillation was carried out for approximately one hour. During this time, the distillate was collected at a rate of roughly one drop per second.

Once approximately 80 mL of toluene had been collected in a graduated cylinder, heating was discontinued, and the reaction flask was allowed to cool to room temperature. An empty 50.0 mL Erlenmeyer flask was weighed, and subsequently re-weighed after transferring the ketal product into it. A small volume of the product (approximately 1.0 mL) was placed into a clean, vacuum-dried NMR tube for analysis. This sample was reserved for proton nuclear magnetic resonance spectroscopy (¹H NMR) spectroscopy to confirm the identity and purity of the ketal product. The remainder of the purified product was weighed, and its mass was recorded for yield calculations.

Period 3: Analysis of ketal

The final phase of the experiment involved the analysis of the ketal using ¹H NMR. From the collected spectrum, characteristic peaks corresponding to the ethyl acetoacetate ethylene ketal (also referred to as fructone) and residual toluene were identified based on their chemical shifts. The areas under the respective peaks were measured to determine the relative abundance of each compound in the final mixture. Using these peak integrations, the percent composition by weight of the desired ketal and the toluene impurity was calculated. From this, the actual mass of pure ketal in the final product was determined. The theoretical yield was calculated based on the initial molar quantities of the starting materials, and the percent yield was derived by comparing the actual and theoretical yields. These values are summarized in the next section (Discussion). In preparation for the subsequent Grignard reaction, all glassware used throughout the synthesis, including the 500 mL three-necked round-bottom flask, 125 mL addition funnel, 250 mL separatory funnel, 100 mL graduated cylinder, distillation condenser, and both 125 mL and 250 mL Erlenmeyer flasks, were thoroughly cleaned, dried, and set aside for reuse.

Experimental details

Period 1: Protection of the Carbonyl Group 

During the protection of ketone synthesis of ethyl acetoacetate ethylene ketal, 100.0 ml of toluene, 25.5 mL of ethyl acetoacetate, 22.3 mL ethylene glycol, 0.2 g of p-toluenesulfonic acid monohydrate, and three boiling chips were added to a 500.0 mL round-bottom flask and was then refluxed in the Dean-Stark apparatus for one hour. After the mixture was refluxed for 1 hour, the heat was then stopped, and the water was allowed to run until the round bottom flask was at room temperature. Then the water flow was stopped and the water from the trap was poured into the waste container. The solution was transferred to a separatory funnel where two visible layers of ether and aqueous was observed. The mixture was extracted with 20.0 mL of cold sodium hydroxide. Then the organic layer was washed with 20.0 mL of deionized water two times and extracted. Then the organic layers were combined and anhydrous magnesium sulfate (MgSO4) was added to dry the solution and get rid of any remaining water. Then the solution was poured through a gravity filter and saved for the next class period. 

Period 2: Isolation of Ethyl Acetoacetate Ethylene Ketal

During the isolation of the ethyl acetoacetate ethylene ketal, the joints of the simple distillation apparatus were greased as with the other apparatus setups. Then the product was added to the three-necked round-bottom flask with some boiling chips and the water flow was turned on and the heating block was set to 40°C, and the mixture was left to distill for an hour. The mixture was distilled at a rate of about one drop per second. Once about 80 mL of toluene was collected in a 100.0 mL graduated cylinder, then the heating mantle was turned off and the round bottom flask was cooled to room temperature. Then an empty 50.0 mL Erlenmeyer flask was weighed and re-weighted with the product in the flask. An NMR tube was washed with acetone and deionized water and was then dried in vacuum. About 1.0 mL or one droplet of our product was dropped into the labeled NMR tube. Then the weight of the ethyl acetoacetate ethylene ketal (fructone) was calculated.

Period 3: Analysis of ketal 

During the analysis of the ketal, the proton nuclear magnetic resonance spectroscopy spectrum of our product was analyzed. The products were identified through signals like the chemical shift of fructone and toluene, as well as finding the area under the peak for both molecules. Then calculated the amount of toluene that was left, if there was any left, and the amount of fructone that was present in our product was calculated. Then using the percent by weight previously calculated, the actual yield of ketal was calculated and the theoretical yield of ketal was also calculated. Then from these two values, the percent yield was found and all values can be found in the Discussion section. All glassware that was needed for the Grignard reaction - which includes 500 mL three-necked flask; 125 mL additional funnel; 250 mL separatory funnel; 100 mL graduated cylinder; condenser (dist); 125 and 250 Erlenmeyer flasks and stoppers - was washed.

## Discussion

Period 1: Protection of the carbonyl group

The initial step of the experiment involved the formation of the ethylene ketal derivative of ethyl acetoacetate. This was achieved through the reaction of the ketoester with ethylene glycol in the presence of the acid catalyst p-toluenesulfonic acid (TsOH), which facilitates the reversible condensation of diols and carbonyl compounds into cyclic ketals. The goal of this step was to selectively protect the more reactive ketone carbonyl group without altering the ester functionality [4].

To initiate the synthesis, stoichiometric calculations were performed to determine the appropriate reagent volumes based on molar equivalents. Ethyl acetoacetate (0.2 mol) was converted using its molar mass and density, yielding 25.5 mL of liquid reagent. Ethylene glycol, used in a 2:1 molar ratio (0.4 mol), was calculated to require 22.3 mL. The theoretical volume of water generated as a byproduct was also determined to be approximately 3.6 mL. These measurements ensured accurate reagent proportions and allowed for monitoring of water removal during the reaction as stated in Table 1.

**Table 1:** Reactants Added to 500mL Three-Necked Flask Summary of reagents and their respective volumes or masses used in the initial reflux step for ketal formation. Ethyl acetoacetate, ethylene glycol, and p-toluenesulfonic acid were combined with toluene and boiling chips for azeotropic distillation.

Reagent	Volume/Mass
Ethyl Acetoacetate	25.5 mL
Ethylene Glycol	22.3 mL
p-Toluenesulfonic Acid	0.2 g
Toluene	100 mL
Boiling Chips	3 chips

The calculated volumes of ethyl acetoacetate and ethylene glycol, along with 100.0 mL of toluene and 0.2 g of TsOH, were introduced into a 500 mL round-bottom flask. To promote even boiling and prevent bumping, three boiling chips were added. The flask was assembled with a Dean-Stark apparatus and subjected to reflux for one hour. During heating, the azeotropic mixture of toluene and water evaporated, condensed in the reflux condenser, and separated in the Dean-Stark trap. The less dense toluene phase recirculated into the flask, while the denser aqueous phase collected in the trap. This continuous removal of water shifted the equilibrium toward ketal formation.

Once the reflux was complete, the reaction was allowed to cool to ambient temperature. The aqueous phase was carefully removed from the trap and discarded. The contents of the flask were transferred to a separatory funnel to facilitate the extraction process. The organic phase, containing the ketal product, was first washed with 20.0 mL of cold sodium hydroxide to neutralize residual TsOH. This was followed by two sequential washes with deionized water to remove inorganic contaminants. After separation, the organic layer was dried using anhydrous magnesium sulfate to eliminate the remaining traces of moisture. The resulting solution was then gravity-filtered to remove the drying agent, and the purified organic phase was stored in preparation for the distillation step.

This portion of the synthesis not only served to selectively protect a functional group but also provided hands-on experience with several foundational techniques in synthetic organic chemistry. These included stoichiometric planning, equilibrium manipulation, reflux with azeotropic distillation, and liquid-liquid extraction, procedures that are routinely employed in both academic and industrial pharmaceutical research.

The purpose of this step was to protect the reactive ketone group in ethyl acetoacetate via the formation of a ketal using ethylene glycol and catalytic TsOH. This reaction is reversible and generates water as a byproduct. To drive the reaction toward completion, water was continuously removed using a Dean-Stark apparatus. As the mixture refluxed, the azeotropic distillation removed water, allowing the equilibrium to favor ketal formation. Once sufficient distillation was observed, the reaction mixture was cooled to room temperature.

Subsequently, the mixture was transferred to a separatory funnel and washed with cold sodium hydroxide to neutralize residual acid, followed by two washes with deionized water. The organic layer was dried using anhydrous MgSO₄, which was added twice to ensure complete removal of water. The dried organic solution was gravity filtered and stored for distillation in the next period.

Period 2: Isolation of ethyl acetoacetate ethylene ketal

The second stage of the synthesis focused on the isolation of the ketal product by removing residual toluene via simple distillation. The previously dried organic phase was transferred to a clean, dry three-necked round-bottom flask equipped for distillation. To ensure proper sealing and safe operation, all joints in the distillation apparatus were carefully greased, and several boiling chips were added to prevent bumping.

The flask was gradually heated using a heating block set to 40°C. Distillation proceeded at a steady rate of approximately one drop per second. Over the course of 1 hour, 65 mL of toluene was collected in a graduated cylinder. While the target recovery volume was approximately 80 mL, distillation was halted slightly early due to a noticeable decline in distillate flow during the final 10 minutes. This partial recovery of toluene may have contributed to the minor residual solvent peaks later observed in the NMR spectrum.

After the distillation process was complete, the flask was allowed to cool to room temperature before handling. To determine the mass of the isolated ketal product, an empty, dry 50.0 mL Erlenmeyer flask was weighed and then re-weighed after the product was transferred into it. The difference in mass between the full and empty flask provided the total mass of the ketal product. Specifically, the mass of the product was calculated to be 20.10 g by subtracting the empty flask weight (39.35 g) from the flask containing the product (59.45 g) as seen in Table 2. Rather than recording this calculation as a standalone expression, this value was then used in subsequent yield calculations and purity assessments.

**Table 2:** Isolation Data Measured values from the isolation step following distillation, including the volume of toluene collected and the weight of the product flask before and after product isolation.

Item	Value
Toluene Collected	65 mL
Empty 50 mL Flask Weight	39.35 g
Flask + Product Weight	59.45 g
Total Mass of Product	20.10 g

To prepare for ¹H NMR spectroscopy, approximately 1.0 mL of the crude product was transferred into a clean and vacuum-dried NMR tube. The remainder of the product was sealed and stored for further analysis. The isolation step provided an opportunity to reinforce distillation skills and emphasized the importance of accurate mass measurements and careful thermal control when handling volatile organic solvents [5].

Period 3: Analysis of ketal via NMR

In the final stage of the synthesis, ¹H NMR spectroscopy was employed to evaluate the composition and purity of the ketal product. The primary objective of this analysis was to quantify the extent of toluene removal and confirm the identity of the protected ethyl acetoacetate derivative. The ¹H NMR spectrum exhibited two prominent signals corresponding to the chemical shifts of the ketal (fructone) and residual toluene. Based on their integration values, a precise determination of component ratios was made. The fructone signal appeared at 1.5 ppm with an integration area of 18.65, while the toluene signal appeared at 2.3 ppm with an integration area of 1.61. These values translated into a mass of 18.65 g for the ketal and 1.61 g for residual toluene. From these values, the composition by weight was calculated to be 92.05% fructone and 7.95% toluene. This confirmed a relatively high purity for the protected product, though the presence of residual toluene suggested that the distillation step could have benefited from an extended heating period.

The theoretical yield calculations were performed assuming complete conversion of 0.2 mol of ethyl acetoacetate to its corresponding ketal product, with a molar mass of 174 g/mol. This gave a theoretical yield of 34.84 g. The actual yield, corrected for product purity, was calculated by multiplying the total product mass (20.10 g) by the fractional purity (92.05%), yielding 18.50 g. Consequently, the percent yield was determined by dividing the actual yield by the theoretical yield and multiplying by 100, resulting in a final yield of 53.09% as summarized in Table 3.

**Table 3:** Proton Nuclear Magnetic Resonance Spectrum of the Ketal Product Proton nuclear magnetic resonance spectrum integration values were used to determine the purity of the ketal product. Chemical shifts and peak areas were translated into component masses and percent composition by weight.

Component	Chemical Shift (ppm)	Integration Area	Mass (g)	Percent by Weight
Fructone (Ketal)	1.5	18.65	18.65	92.05%
Toluene	2.3	1.61	1.61	7.95%

In summary, the NMR analysis confirmed the successful formation of the protected ketal with moderate yield and limited solvent contamination. The presence of toluene, although minor, highlighted the importance of careful solvent removal in reaction workups. This step reinforced the critical role of analytical techniques such as NMR in assessing both product identity and purification efficiency in multistep organic synthesis [3].

## Conclusions

The following reactants - toluene, ethyl acetoacetate, ethylene glycol, and p-toluenesulfonic acid - are refluxed in the Dean-Stark apparatus for 1 hour. In a separatory funnel, the reaction mixture is washed with cold sodium hydroxide and deionized water. Anhydrous MgSO4 is used to dry the solution and a gravity filtration is used to collect our product. Toluene is removed from the former product using a simple distillation apparatus. The temperature is monitored in order not to distill the product along with the toluene. The final product weight is 20.1 g of fructose. A 1.0 mL sample of the product is analyzed in an ^1^H NMR spectrum. The spectroscopy showed the presence of 92.05% fructose and 7.95% toluene in the final product. The theoretical yield of the ketal is 34.84 g and the actual yield is 18.5 g ketal. The percent yield is calculated to be 53.10%. Beyond its pedagogical value, this synthesis step reflects core processes in medicinal chemistry and pharmaceutical manufacturing. The ability to selectively protect functional groups, purify intermediates, and analyze product composition mirrors the challenges and precision required in drug design and production. The presence of minor toluene contamination (7.95%) underscores the need to optimize purification protocols in equilibrium-limited reactions, where even small residual solvents can affect downstream applications. Overall, the techniques employed closely simulate real-world workflows in pharmaceutical development, where intermediate control and structural verification are essential. This experiment not only provided technical training but also emphasized the broader relevance of these methods in preparing complex, biologically active molecules.

This synthesis reinforced the foundational concept of functional group protection, demonstrating how masking reactive sites enables more selective downstream transformations. The procedure illustrated the importance of precise stoichiometric planning, careful thermal control, and accurate analytical validation in achieving product purity. The observed yield and minor solvent contamination emphasized the value of optimizing purification conditions, especially in reactions that depend on equilibrium dynamics. Most importantly, the techniques applied here mirror real-world workflows used in pharmaceutical development, where intermediate control and structural verification are essential. This experiment not only provided technical training but also emphasized the broader relevance of these methods in preparing complex, biologically active molecules.
